# 2165. Assessing the Impact of Meropenem Exposure on Ceftolozane/tazobactam-resistance Development in *Pseudomonas aeruginosa* using *in vitro* Serial Passage

**DOI:** 10.1093/ofid/ofad500.1788

**Published:** 2023-11-27

**Authors:** Aliaa Fouad, Samantha Nicolau, Pranita Tamma, Patricia J Simner, David P Nicolau, Christian M Gill

**Affiliations:** Hartford Hospital, Farmington, Connecticut; Sanofi, Boston, Massachusetts; Johns Hopkins School of Medicine, Baltimore, MD; Johns Hopkins School of Medicine, Baltimore, MD; Hartford Hospital, Farmington, Connecticut; Hartford Hospital, Farmington, Connecticut

## Abstract

**Background:**

Patients infected with *Pseudomonas aeruginosa* with difficult-to-treat resistance are likely to receive meropenem (MEM) as an empiric therapy before escalation to broader agent such as ceftolozane/tazobactam (C/T) after antimicrobial susceptibility data are available. We assessed if pre-exposure to MEM impacted the development of C/T-resistance upon C/T exposure.

**Figure:**
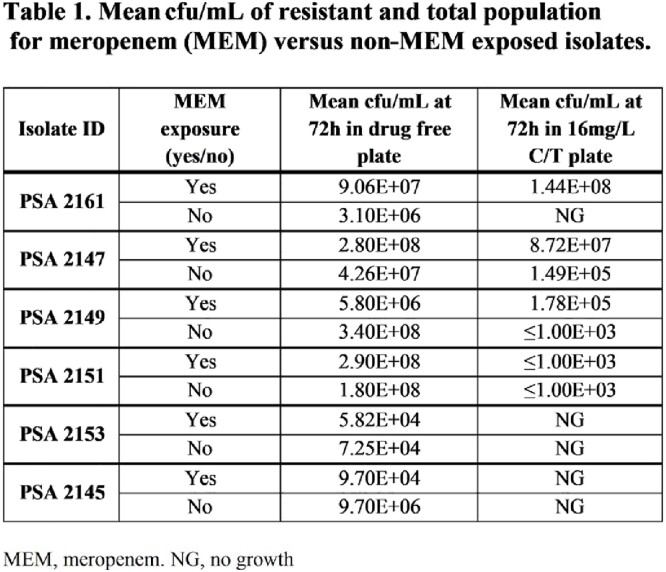

**Methods:**

Six clinical *P. aeruginosa* isolates were assessed. Isolates were exposed to 16 mg/L MEM concentration for 72h, interrupted by wash step every 24h to avoid drug degradation and antibiotic carryover. Then, isolates were serially passaged in the presence of C/T as two groups: MEM-exposed group inoculated with MEM pre-exposed isolates and non-MEM control group. Three consecutive passages took place at a C/T concentration of 10 mg/L for 72h. After 24 h intervals, samples were serially diluted, plated onto Mueller Hinton agar, and incubated to quantify bacterial densities (log_10_ cfu/mL). Samples were plated on drug free and drug containing agar (C/T concentration 16 mg/L) where growth on C/T agar indicated resistance development. Resistant population was calculated by dividing the cfu/mL on C/T containing plates by the cfu/mL on drug free agar (total population). Percentage of C/T-resistant population was compared between MEM-exposed and MEM non-exposed passages of each isolate.

**Results:**

At 72 h, resistant populations were detected in 4/6 isolates (**Table 1**). In three isolates, MEM exposure significantly increases the prevalence of resistance development against C/T; the percentage of resistance population for the three isolates were 100%, 31%, and 3% for the MEM-exposed versus 0%, 0.35%, and ≤0.0003% in the unexposed groups. One isolate had similar percent resistant population at 72 h between groups (≤0.003 and ≤0.005%). The remaining isolates showed no development of resistance in either group.

**Conclusion:**

Previous MEM exposure may pre-dispose to C/T resistance development upon exposure limiting therapeutic utility. Resistance may be a result of the stress exposure or molecular level mutations conferring cross-resistance. Further *in vivo* and clinical data are needed to further assess the clinical implications of these findings.

**Disclosures:**

**Samantha Nicolau, PhD**, Sanofi Pasteur: full time research scientist **Patricia J. Simner, PhD**, Affinity Biosensors: Grant/Research Support|BD Diagnostics: Advisor/Consultant|BD Diagnostics: Grant/Research Support|Entasis: Advisor/Consultant|GeneCapture: Stocks/Bonds|Merck: Advisor/Consultant|OpGen Inc: Board Member|OpGen Inc: Grant/Research Support|OpGen Inc: Honoraria|Qiagen Sciences Inc: Advisor/Consultant|Qiagen Sciences Inc: Grant/Research Support|Shionogi Inc: Advisor/Consultant|T2 Biosystems: Grant/Research Support **David P. Nicolau, PharmD**, Allergan: Advisor/Consultant|Allergan: Grant/Research Support|Cepheid: Advisor/Consultant|Cepheid: Grant/Research Support|Merck: Advisor/Consultant|Merck: Grant/Research Support|Pfizer: Advisor/Consultant|Pfizer: Grant/Research Support|Shionogi: Advisor/Consultant|Shionogi: Grant/Research Support|Tetraphase: Advisor/Consultant|Tetraphase: Grant/Research Support|Venatorx: Advisor/Consultant|Venatorx: Grant/Research Support|Wockhardt: Advisor/Consultant|Wockhardt: Grant/Research Support **Christian M. Gill, PharmD**, Cepheid: Grant/Research Support|Entasis therapeutics: Grant/Research Support|Everest Medicines: Grant/Research Support|Shionogi: Grant/Research Support

